# TGF-β–driven T-cell exclusion in ovarian cancer: single-cell and spatial transcriptomic views of immune low-response states

**DOI:** 10.3389/fimmu.2025.1698088

**Published:** 2025-10-17

**Authors:** Jiang He, Jun Tao, Yu Zhou, Hongjian Li, Wenqi Feng, Yongqiang Xu

**Affiliations:** ^1^ Yibin Institute of Traditional Chinese Medicine, Yibin, Sichuan, China; ^2^ Yibin Hospital of Traditional Chinese Medicine, Yibin, Sichuan, China

**Keywords:** ovarian cancer, TGF-β signalling, T-cell exclusion, single-cell RNA/ATAC, spatial transcriptomics

## Abstract

Epithelial ovarian cancer (EOC) remains a lethal epithelial malignancy. Immune-checkpoint inhibitors have entered management for recurrent/metastatic disease; yet durable benefit is confined to a subset, reflecting TGF-β–conditioned stromal barriers and organised T-cell exclusion. In this review we summarise advances from single-cell RNA and ATAC profiling and spatial transcriptomics that resolve fibroblast, tumour and immune programmes linked to TGF-β signalling, and appraise translational opportunities spanning selective pathway modulation, checkpoint combinations and spatial biomarkers. We also discuss enduring challenges—including site-specific heterogeneity across adnexal, omental and peritoneal niches, limited assay standardisation and a scarcity of predictive metrics—that temper implementation. By integrating TGF-β–informed readouts (e.g., INHBA^+^ cancer-associated fibroblast burden, periostin/fibronectin indices, MHC-I status and CD8–tumour distances) with PD-1–based regimens and TGF-β-axis agents (ALK5 inhibitors, Activin A neutralisation, NOX4-directed reprogramming), emerging strategies aim to restore antigen presentation, improve lymphocyte access and remodel tumour–stroma interfaces. Our synthesis provides an appraisal of the evolving landscape of TGF-β–informed precision immuno-oncology in ovarian cancer and outlines pragmatic standards and avenues for clinical translation. We hope these insights will assist researchers and clinicians as they endeavour to implement more effective, individualised regimens.

## 1 Introduction

Epithelial ovarian cancer remains the most lethal gynecologic malignancy, with high-grade serous ovarian carcinoma (HGSOC) accounting for the majority of deaths and displaying pronounced genomic instability and tissue-site heterogeneity that complicate immune control (1–3). Single-cell and spatially resolved studies demonstrate that immune activation and suppression can segregate across intraperitoneal niches in HGSOC, with microenvironmental context shaping recognition and escape (4–7). Immunotherapy with immune-checkpoint inhibitors has produced limited and variable benefit in unselected ovarian cancer populations, underscoring the need to resolve mechanisms of immune failure at cellular and spatial resolution.

Transforming growth factor-β1/β2/β3 from tumor cells, CAFs and Tregs drive exclusion via TGFBR1/ALK5-SMAD2/3 plus non-SMAD (p38/ERK/PI3K) arms, while CAF-derived Activin A (INHBA) engages ACVR1B/ACVR2 to phenocopy these suppressive effects and blunt PD-(L)1 responses (8–11). Foundational work in urothelial and colorectal cancer showed that TGF-β–dependent stromal activation confines effector T cells to peritumoral territories and that dual blockade of TGF-β and PD-(L)1 can restore intratumoral T-cell access (12–15). Consistent with these principles, pan-cancer analyses link high TGF-β activity to immune-excluded phenotypes and resistance to checkpoint inhibition.

Evidence specific to ovarian cancer supports a TGF-β–conditioned, stromal-dominated immune low-response state. Integrated digital pathology and transcriptomics identified TGF-β–driven loss of antigen presentation and fibroblast activation as mediators of T-cell exclusion in ovarian tumors, with reduced MHC-I on cancer cells and desmoplastic barriers that hinder infiltration (16–18). Single-cell and spatial profiling of HGSOC further resolve site-specific immune ecosystems, revealing that tumors with copy-number–driven evolution can exhibit elevated TGF-β signaling alongside naïve or memory-skewed T-cell compartments and limited effector access. Spatial atlases also document marked heterogeneity of tumor-infiltrating T cells and their neighborhood relationships with stromal and malignant cells, providing a structural substrate for immune exclusion (19–21). Within the stromal compartment, immunomodulatory cancer-associated fibroblast subsets, including INHBA^+^ CAFs that enforce SMAD2-dependent PD-L1 expression and regulatory T-cell differentiation, exemplify TGF-β–linked suppressive circuits in advanced ovarian cancer. Preclinical work in HGSOC models shows that concurrent targeting of TGF-β and PD-L1 can enhance antitumor immunity, consistent with a causal role for TGF-β in therapeutic nonresponse.

Single-cell RNA sequencing, single-cell chromatin and spatial transcriptomic technologies now permit direct quantification of TGF-β pathway activity, fibroblast and extracellular-matrix programs, and ligand–receptor interactions that organize T-cell exclusion in ovarian cancer (22–24). By integrating these modalities, it is feasible to define reproducible immune low-response phenotypes, map their stromal drivers, and derive composite biomarkers and testable interventions. The purpose of this review is to synthesize single-cell and spatial transcriptomic evidence on TGF-β–driven T-cell exclusion in ovarian cancer, delineate mechanistic links between signaling and stromal remodeling, and outline diagnostic and therapeutic implications for risk stratification and treatment design.

## 2 Single-cell and spatial phenotype of TGF-β–conditioned immune low-response in ovarian cancer

Single-cell and spatial studies in high-grade serous ovarian carcinoma (HGSOC) converge on a reproducible immune–stromal state in which transforming growth factor-β (TGF-β) signaling coincides with peritumoral confinement of effector T cells, reduced antigen presentation, and desmoplastic remodeling (25–30).

**Figure 1:**
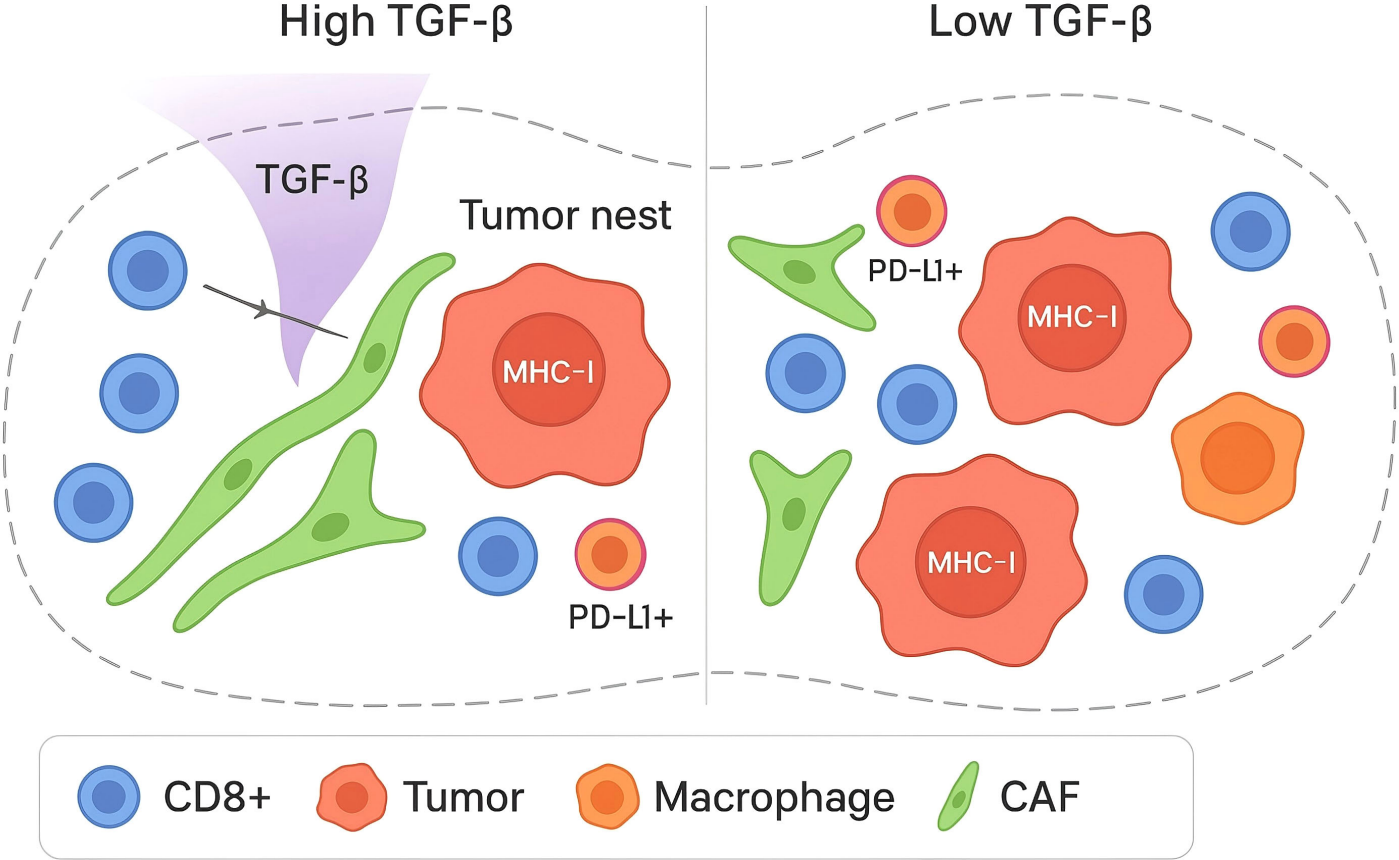
TGF-β–driven T-cell exclusion in the ovarian cancer microenvironment.

In HGSOC, multi-site single-cell and multiplex imaging analyses show that anatomical location and mutational processes stratify immune phenotypes (30–32). Tumors bearing fold-back inversions exhibit elevated TGF-β pathway activity with immune-excluded architectures populated by naïve/stem-like and memory-skewed T-cell compartments, whereas homologous-recombination-deficient tumors display more differentiated dysfunctional CD8+ states (33, , 12). These patterns are quantified by nearest-neighbor distance (centroid-to-centroid μm after nuclei segmentation; k-d tree), tumor–stroma interface length (contiguous boundary μm by skeletonization), and CAF ‘corridor’ width (fibronectin/α-SMA–positive bands via binary morphology). As shown in **Figure 1**, these site and genotype-linked patterns are quantified by nearest-neighbor distances between CD8+ T cells, PD-L1+ cancer cells, and PD-L1+ macrophages, indicating reduced effector proximity in TGF-β–high contexts.

Single-cell atlases further resolve T-cell heterogeneity across ovarian and omental foci. Ovarian lesions often show ‘cold’ states with Tregs and dysfunctional T cells, while omentum harbors bystanders; canonical markers/niches include TCF1^+^ stem-like CD8 (TCF7, SLAMF6) perivascular/TLS-adjacent, terminally exhausted CD8 (PD-1, TOX, TIM-3) at margins, bystander CD8 (CD39^-^) in omentum, and Tregs (FOXP3, CTLA-4, TIGIT) in CAF-rich rims (34–37). These features are consistent with a TGF-β–conditioned, stromal-dominated immune low-response.

Fibroblast programs are central to this phenotype. A TGF-β–driven cancer-associated fibroblast (CAF) subset identified by scRNA-seq adversely associates with outcome and expresses TGF-β pathway and EMT-linked effectors (38–40). Complementing this, INHBA+ (Activin A–producing) CAFs enforce SMAD2-dependent PD-L1 expression and promote regulatory T-cell differentiation, providing a direct cellular mechanism for immunosuppression within advanced ovarian tumors.

Operational markers used in this review to define the TGF-β–conditioned immune low-response state are shown in **Table 1**. Spatial proteogenomic profiling in ovarian cancer supports these single-cell inferences: immune-excluded regions are enriched for Tregs and fibronectin-rich stroma, whereas diffuse, tumor-proximal immune niches exhibit higher PD-L1/IDO1 and activated lymphocyte markers (41–43). These observations align with a model in which TGF-β-conditioned fibroblast matrices and checkpoint-ligand geography jointly restrict productive cytotoxic engagement.

**Table 1:** Operational features and readouts of a TGF-β–conditioned immune low-response state in ovarian cancer.

Phenotypic component	Single-cell transcriptomic indicators	Ligand–receptor/chromatin indicators	Spatial readout	Interpretive note
Fibroblast TGF-β program (myofibroblastic/TGF-β–driven CAFs; INHBA+ CAFs)	High COMP, THBS1, TGFBI, LTBP2, SKIL; INHBA/INHBB; COL10A1/COL11A1/MMP2/MMP14	TGFB1–TGFBR1/2; INHBA–ACVR1B/ACVR2; SMAD2/3 target-gene activation; PD-L1 on CAFs	Desmoplastic rim at tumor–stroma borders; fibronectin-rich corridors	Stromal barrier formation; Treg induction; checkpoint-ligand provision
Tumor-intrinsic TGF-β activity and antigen presentation status	EMT and SMAD target signatures; lower HLA-A/B/C, B2M in subsets	PD-L1 (CD274) upregulation; diminished MHC-I processing genes (e.g., TAP1/2)	Increased CD8+–tumor cell distances; CD68+PD-L1+ interfaces	Impaired CD8+ recognition and effector engagement
T-cell compartment in immune-low states	Naïve/meory-skewed CD8+/CD4+; fewer tumor-specific exhausted CD8+ in selected sites; Treg enrichment	PD-1 (PDCD1) on T cells with limited cognate antigen; ICOS/ICOSL low in tolerized niches	Bystander-rich omental foci; paucity of intraepithelial CD8+; sparse TLS	Limited priming and trafficking into tumor nests
Myeloid/DC context	Migratory LAMP3+ DC; reduced cDC1; macrophage PD-L1	PD-1/PD-L1 ligation zones at margins; TGFB1–TGFBR signaling in stroma	Macrophage layers lining tumor borders; stromal “corridors”	Sustained checkpoint signaling at interfaces; restricted infiltration

These single-cell and spatial criteria delineate an ovarian cancer ecosystem in which TGF-β–responsive fibroblast matrices, altered antigen presentation, and checkpoint-dominated contact zones converge to produce immune exclusion; this phenotype maps onto the broader TGF-β barrier framework defined in other indications and provides tractable readouts for risk stratification and therapeutic testing.

## 3 Mechanistic links between TGF-β signaling, stromal remodeling, and T-cell exclusion

Transforming growth factor-β orchestrates a fibroblast-centered program that remodels the extracellular matrix and establishes spatial barriers to effector T-cell access. In multiple solid tumors, stromal TGF-β activity correlates with immune-excluded architectures, and experimental inhibition of TGF-β restores intratumoral T-cell penetration when combined with PD-(L)1 blockade, indicating that TGF-β–dependent stromal activation is a proximal cause rather than an epiphenomenon of exclusion (44–46). Mechanistically, TGF-β/SMAD signaling in cancer-associated fibroblasts (CAFs) induces contractile myofibroblastic states and upregulates matrix constituents and modulators—collagens, fibronectin, versican, thrombospondins, latent TGF-β–binding proteins—together with crosslinking and alignment programs that increase stiffness and reduce interstitial porosity, thereby constraining lymphocyte trafficking (47–49). Ovarian tumor stroma exemplifies these dynamics: TGF-β1–induced periostin in activated fibroblasts promotes desmoplastic remodeling and malignant cell motility, reinforcing matrix-rich interfaces at tumor borders that are unfavorable to T-cell ingress (50–52). These observations align with pan-cancer ECM signatures linked to TGF-β and poor ICI outcomes; chemokine circuits (CXCL12–CXCR4, CCL2–CCR2, TGF-β–induced CXCLs) cooperate with aligned collagen/fibronectin to confine cells peritumorally.

In ovarian cancer, TGF-β shapes immunoregulation beyond its effects on physical barriers. Integrated digital pathology and transcriptomics show that TGF-β correlates with diminished tumor-cell antigen presentation and fibroblast activation in T-cell–excluded tumors, indicating concurrent defects in recognition and access (53–55). Spatial proteogenomic profiling further demonstrates that fibronectin-rich stromal territories with regulatory T-cell enrichment co-localize with immune-excluded niches, whereas areas with diffuse tumor–immune proximity display higher antigen-presentation markers and checkpoint expression, consistent with segregation of suppressive matrix from effective cytotoxic engagement (56–58). At the cellular level, INHBA^+^ CAFs release bioactive Activin A via furin-mediated prodomain cleavage; ACVR1B/ACVR2→SMAD2 signaling in CAFs induces PD-L1 and Treg programs, sustaining exclusion even when T cells reach the margin (59–61). Tumor-derived TGF-β1 promotes CAF differentiation and metastatic competence, supplying ligand to maintain these stromal and immunosuppressive circuits. Taken together, these data delineate a convergent mechanism whereby TGF-β–responsive fibroblast programs generate an ECM-defined barrier, reduce antigen visibility, and install local checkpoint ligation, yielding a T-cell–excluded, immune low-response state.

## 4 Diagnostic trajectory and risk stratification from single-cell and spatial readouts

Diagnostic evaluation of immune low-response ovarian cancer should progress from discovery-grade single-cell and spatial assays to deployable, site-aware risk stratification that quantifies TGF-β–conditioned stromal programs, antigen-presentation deficits, and the geometry of tumor–immune contacts. In high-grade serous ovarian carcinoma (HGSOC), spatial transcriptomics shows that discrete malignant subclones occupy distinct neighborhoods and engage defined stromal and immune partners, indicating that clone-specific ligand–receptor circuits partly encode the degree and pattern of lymphocyte access; these features are directly measurable in tissue and link to outcome-relevant biology (62–65). Integrative multi-omic mapping across 160 tumor sites further demonstrates that mutational processes and anatomic location co-determine immune states, with fold-back inversion–bearing tumors exhibiting elevated TGF-β signaling, T-cell exclusion, and naïve/memory-skewed T-cell compartments—an axis that plausibly marks a TGF-β–high risk group (66–68). In parallel, spatial proteogenomic profiling in ovarian cancer separates diffuse tumor–immune interdigitation from focal immune niches; the former co-localizes with higher PD-L1/IDO1 and other immunotherapy targets, while focalized macrophage-rich niches (CD163^high^) associate with preliminarily worse outcomes, supporting the use of neighborhood metrics rather than bulk density alone for risk definition (69–71). These observations provide a rationale to define a composite “TGF-β–conditioned exclusion” classifier that integrates four orthogonal readouts: a stromal/CAF activity index, an antigen-presentation index, a spatial interaction index, and a contextual genetic index.

For the stromal/CAF activity index, single-cell–derived markers of TGF-β–responsive fibroblasts can be translated to practical surrogates. INHBA^+^ cancer-associated fibroblasts drive SMAD2-dependent PD-L1 expression and promote regulatory T-cell differentiation in advanced ovarian cancer, nominating INHBA protein/RNA and CAF-PD-L1 as tissue surrogates of TGF-β superfamily–linked immune suppression (72–74). Periostin-rich matrices—induced through integrin/NF-κB and TGF-β2 signaling—track with macrophage recruitment and fibroblast activation in ovarian cancer and can serve as desmoplastic sentinels measurable on archival formalin-fixed tissue (75–78). For the antigen-presentation index, HR-deficient contexts show enhanced immunosurveillance, whereas HR-proficient tumors display compartmentalization; incorporate MHC-I surrogates with HRD and note: MHC-I loss via B2M truncation, HLA LOH, or IFN–JAK/STAT defects; readouts—IHC (HLA-A/B/C; B2M 0–3+ rubric) and copy-number flags (79–81). The spatial interaction index should quantify nearest-neighbor distances and interface lengths between CD8+ T cells, PD-L1+ tumor/myeloid cells, and CAF corridors, because diffuse tumor–immune mixing versus focal or peritumoral restriction carries distinct therapeutic implications in ovarian cancer (82–84). The contextual genetic index should register fold-back inversions and other copy-number–driven processes that associate with high TGF-β activity and immune exclusion, as these events stratify immunologic phenotypes across intraperitoneal sites.

Assay implementation can follow a tiered path compatible with routine specimens. Discovery-level single-cell and spatial transcriptomic platforms define cell states, ligands, and receptor topologies; these can be down-translated to validated multiplex protein imaging on formalin-fixed sections. High-dimensional imaging methods such as multiplexed ion-beam imaging by time-of-flight (MIBI-TOF) have demonstrated reproducible, quantitative annotation of clinically relevant cell states in archival tissues and provide a route to standardize spatial scoring rules across centers (85–87). For institutions without high-plex capacity, constrained surrogate panels can approximate the composite score by combining INHBA/α-SMA/fibronectin/periostin with PD-L1, HLA-I components, and pan-T-cell markers, quantified with pre-specified adjacency metrics. Spatial risk assignments should be site-aware, because adnexal, omental, and peritoneal foci exhibit different immune architectures under the same patient-level genotype, and because subclones within a lesion can preferentially associate with fibroblasts or CXCL9^+^ macrophages. Where available, radiogenomic bridges that correlate spatial transcriptomic phenotypes with computed tomography features can facilitate non-invasive stratification and longitudinal monitoring.

In terms of clinical use, the composite classifier should separate at least two actionable risk states. A TGF-β–dominant, CAF-rich, immune-excluded state—scored by high INHBA/periostin/fibronectin, low MHC-I, long CD8^+^–tumor distances, macrophage/CAF border interfaces, and FBI-like genomic context—would be predicted to benefit from strategies that decompress or reprogram stroma and restore access, including consideration of TGF-β pathway targeting layered onto PD-(L)1 where feasible. The biological basis rests on studies in other solid tumors in which stromal TGF-β blockade restored intratumoral T-cell access and synergized with PD-(L)1 inhibition, supporting the face validity of this state as a barrier phenotype (88–90). A comparatively inflamed state—characterized by diffuse tumor–immune contact, TLS/B-cell aggregates, and intact antigen presentation—could be triaged toward checkpoint-based regimens or trials emphasizing antigen-presentation and costimulation, with ovarian data showing that TLS and diffuse tumor–immune interactions track with favorable immune targets and improved prognostic signals (26, 91, 92). Prospective validation should predefine analytic thresholds, ensure inter-assay concordance between discovery and surrogate panels, and embed multi-site sampling to avoid misclassification by local ecology; however, the current body of single-cell and spatial evidence already delineates measurable features that can be operationalized to forecast T-cell access, checkpoint-ligand geography, and TGF-β–linked stromal risk in ovarian cancer.

## 5 Therapeutic strategies and future directions

Therapeutic development for a TGF-β–conditioned, immune-excluded state in ovarian cancer should prioritize combinations that restore intratumoral CD8^+^ T-cell access while minimizing pathway-wide toxicities. Convergent preclinical work demonstrates that stromal TGF-β activity enforces peritumoral confinement of effector T cells and that simultaneous inhibition of TGF-β and PD-(L)1 converts exclusion into productive antitumor immunity; these data provide a mechanistic basis for layered regimens in TGF-β–high ovarian tumors identified by single-cell and spatial criteria (93–95). However, the pleiotropic roles of TGF-β mandate selective approaches. Strategies that confine pathway blockade to dominant immunoregulatory sources or nodes are attractive—for example, antibody targeting of GARP: TGF-β1 complexes to restrict neutralization to regulatory T-cell–derived ligand, or context-adapted ALK5 inhibition administered in intermittent schedules to mitigate toxicity—both supported by translational and early clinical literature.

Ovarian-specific stromal targets emerging from single-cell and spatial profiling nominate tractable entry points. INHBA^+^ (Activin A–producing) cancer-associated fibroblasts upregulate PD-L1 via SMAD2-dependent signaling and drive regulatory T-cell differentiation; neutralization of Activin A attenuates disease and remodels the immune–stromal compartment in ovarian models, indicating a rational partner for PD-(L)1 or costimulatory strategies in INHBA-high states (96–98). Periostin-rich matrices induced through integrin/NF-κB and TGF-β2 signaling associate with macrophage recruitment, fibroblast activation, and metastatic competence in epithelial ovarian cancer, supporting periostin or upstream integrin blockade as stroma-decompressing adjuncts in exclusion phenotypes. Because CAF-driven mechanics and chemokine circuits are central to immune geography, pharmacologic reprogramming rather than indiscriminate depletion is preferred; inhibition of NOX4, a TGF-β–linked driver of myofibroblastic states, overcomes CAF-mediated CD8^+^ T-cell exclusion and potentiates checkpoint efficacy across models, justifying evaluation in ovarian desmoplastic contexts (99–101). Beyond ligand- or matrix-focused interventions, clinically advanced TGF-β receptor I (ALK5) inhibitors offer near-term feasibility for combination regimens. Vactosertib has shown signals of activity in combination with pembrolizumab in microsatellite-stable colorectal cancer and favorable safety in hematologic malignancy when paired with an immunomodulatory backbone, motivating disease-adapted trials in ovarian cancer cohorts molecularly enriched for TGF-β–conditioned exclusion.

Implementation should be explicitly biomarker-driven and site-aware. As outlined by spatial and single-cell evidence, risk assignment can integrate a stromal/CAF activity index (e.g., INHBA, periostin, fibronectin), an antigen-presentation index, and quantitative interaction metrics (nearest-neighbor distances and interface lengths among CD8^+^ T cells, PD-L1^+^ tumor/myeloid populations, and CAF corridors). Pharmacodynamic endpoints: ≥20–30% shortening of CD8^+^–tumor distances, ≥30% reduction of continuous macrophage/CAF–tumor interfaces, and ≥1-grade HLA-I upshift with emergence of tumor–immune interdigitation; biopsy at baseline and ~2–4 weeks on-treatment. Standardized multiplex tissue imaging permits these readouts on archival formalin-fixed sections; MIBI-TOF has demonstrated reproducible, quantitative annotation of clinically relevant cell states and can anchor cross-center harmonization of spatial metrics (102–105). Given the heterogeneity of adnexal, omental, and peritoneal ecosystems, protocols should mandate multi-site sampling and predefine adjudication rules when spatial phenotypes diverge within a patient. In inflamed tumors with B-/T-cell aggregates, preserve/induce TLS: emergence and maintenance require CXCL13 and LTα/β; dose stromal modulation intermittently and tissue-sparing to avoid TLS disruption, pairing with DC/costimulatory support.

Future studies should prospectively test a tiered combination schema aligned to spatially measured biology. In a TGF-β–dominant, CAF-rich exclusion state, a backbone of PD-(L)1 with a TGF-β–axis agent selected to the dominant source (e.g., Activin A neutralization in INHBA-high CAF contexts or ALK5 inhibition with intermittent dosing) can be layered with CAF reprogrammers such as NOX4 inhibitors; in comparatively inflamed, TLS-rich states, emphasis can shift toward antigen-presentation and costimulation with stromal restraint. Trial designs should incorporate adaptive stopping rules tied to on-treatment engagement of the intended axis and include safety guardrails informed by the historical toxicity profile of TGF-β inhibitors (cutaneous events, gastrointestinal symptoms, and rare cardiotoxicity), with dosing schedules and patient selection optimized to minimize non-target immunologic perturbation. These principles convert single-cell and spatial readouts into actionable therapeutic logic: deconstrain access when T cells are present but excluded, restore recognition when antigen visibility is limited, and preserve organized immune niches when they emerge under therapy.
